# Hypoparathyroidism Revealed by Unsuccessful Anti-epileptic Therapy

**DOI:** 10.7759/cureus.54200

**Published:** 2024-02-14

**Authors:** Raja Arrab, Youssef Benchehab, Fadila Guessous, Nezha Dini

**Affiliations:** 1 Department of Pediatrics, Mohammed VI International University Hospital, Mohammed VI University of Health Sciences (UM6SS), Casablanca, MAR; 2 Department of Biological Sciences, Faculty of Medicine, Mohammed VI University of Health Sciences (UM6SS), Casablanca, MAR; 3 Department of Pediatrics, Mohammed VI University of Health Sciences (UM6SS), Casablanca, MAR

**Keywords:** children, phosphocalcium metabolism, hypocalcemia, seizure, hypoparathyroidism

## Abstract

Pediatric hypoparathyroidism is an uncommon endocrine disease that can be either isolated or syndromic. It occurs when the secretion of parathormone is insufficient to maintain normal levels of ionized calcium. Patients with hypoparathyroidism can exhibit cerebral calcifications and metabolic disorders, and the severity of such features is inversely correlated with hypocalcemia.

We report a case of a 13-year-old patient who was initially diagnosed with epilepsy by another medical team two years before her admission to our hospital and who was subjected to oral valproate therapy. The anti-epileptic therapy proved to be unsuccessful even with increasing doses. The diagnosis was corrected when we performed adequate biological investigations. This case is underlying the importance of the electrolytes profile, especially the serum phosphocalcic test, in the management of patients with new onset or recurrent epileptic seizures.

## Introduction

Hypoparathyroidism is a rare endocrine disease that can affect adults and children from the neonatal period, and it can be either isolated or syndromic. Hypoparathyroidism results from deficiency or resistance to parathormone, and it is associated with biological manifestations, such as hypocalcemia and hyperphosphatemia with low parathormone serum levels. Moreover, the severity of the hypoparathyroidism symptoms, including seizures, laryngospasm, and heart rhythm disorder, correlates with the hypocalcemia level [1]. Usually, hypocalcemia related to hypoparathyroidism is not often symptomatic [1]. 

Here in, we report a case of a 13-year-old patient who exhibited seizure as a first manifestation of hypoparathyroidism.

## Case presentation

We report a case of a 13-year-old female patient who was admitted to the pediatrics department upon focal seizure episodes. The patient was previously healthy, and she was delivered on term with a normal adaptation to extrauterine life and had normal psychomotor and growth development. There was no history of epilepsy in the family nor autoimmune disease context. 

The symptomatology began by the age of 11 years with right upper limb myoclonus, and then, seizures worsened with daily recurrence. The patient was diagnosed with epilepsy and started on oral valproate treatment. No improvement was reported in her symptoms. It is important to note that the patient was not treated by us during the first year from the onset of the disease and that we don’t have her medical records. 

Upon initial examination, the patient was conscious but manifested a slowing mental reaction. No dysmorphic feature was observed, and she had muscle weakness, especially on the right upper limb. The rest of the clinical exam was normal. 

**Figure 1:**
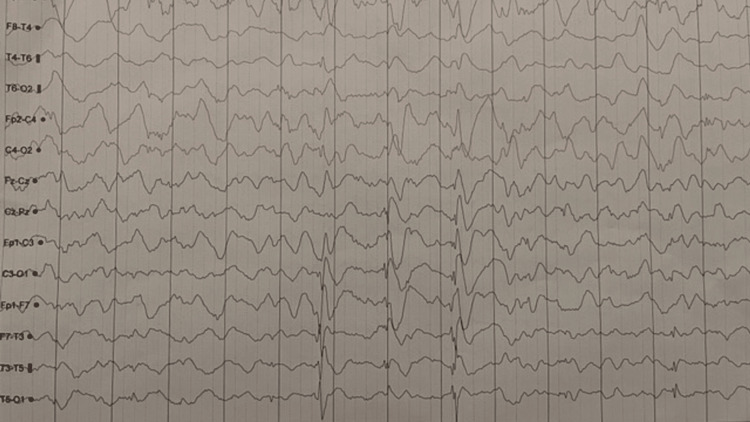
Electroencephalography: frontal spikes discharge with left temporoparietal slow-waves

The laboratory results showed normal blood count and normal levels of glucose. The serum electrolyte profile revealed a low level of calcium at 44 mg/L (84-102 mg/L), a hyperphosphatemia at 92.3 mg/L (28-48 mg/L), and a low parathormone level at 3.6 ng/L (15-65 ng/L). The level of magnesium was low at 14.1 mg/L. The urea and creatinine tests were normal; the vitamin D level was 20.9 ng/mL, the urine calcium level was 57 mg/L, and the urine creatinine level was 981.8 mg/L. The MRI was normal, with no signs of calcifications or other abnormalities. The electroencephalography shows that frontal spikes discharge with left temporoparietal slow-waves (Figure 1).

The patient received intravenous calcium gluconate and magnesium. Oral calcium and vitamin D supplementation were then initiated. The serum calcium levels returned to normal, and the frequency of seizures decreased considerably. The patient reached a seizure-free status with considerable overall physical condition improvement.

## Discussion

Hypoparathyroidism is a rare endocrine disease that results from a deficiency or resistance to parathormone. Biological manifestations include hypocalcemia, hyperphosphatemia, and low parathormone serum level [1]. In children, hypoparathyroidism is more likely to be genetic than acquired; however, it does not necessarily imply a diagnosis in the neonatal period, as it can become symptomatic later in childhood [2]. Because of the high calcium requirement, hypoparathyroidism is more likely to be symptomatic in children than in adults.

In the context of hypoparathyroidism, seizures are mainly explained by hypocalcemia, which lowers the seizure’s threshold [3]. While seizures can occur at any age, it is important to highlight that two periods are at high risk of symptomatic hypocalcemia in children, early childhood and adolescence, because of increased calcium needs during these stages of life [4]. Cerebral calcifications can occur in phosphocalcic metabolism disorder, which is defined as Fahr’s syndrome [5]. It should be distinguished from Fahr disease, which does not contain phosphocalcic metabolism abnormalities. The mechanism of seizures is explained by reduced excitability, an increased neural transmission, and neuromuscular excitation due to hypocalcemia, which can also lead to an increase in hippocampal neurons to epilepsy [6].

Our patient was managed as an epileptic case for about one year before her admission to our hospital. No blood test was performed initially. It seems important to focus on the role of the electrolyte profile in the diagnostic approach of seizures in children, even in the context of febrile seizures. Fever can reveal underlying phosphocalcic abnormality associated with increasing needs for calcium [7].

An oral anti-epileptic treatment was given to our patient without any improvement, even with gradually increased doses. The management of epileptic seizures in the context of hypoparathyroidism is mainly based on phosphocalcic supplementation. Actually, despite the presence of cortical calcifications, anti-epileptic therapy does not improve the seizures’ outcomes [8]. Moreover, anti-epileptic drugs can affect phosphocalcic metabolism, considering their inhibitory effect on the intestinal absorption of calcium. Other studies indicate that patients with hypoparathyroidism may achieve good outcomes for seizures without a need for anti-epileptic disease treatment [9]. In the study of Modi et al., it was possible to stop anti-epileptic drugs, under strict criteria, in 71% of patients with idiopathic hypoparathyroidism with a significant improvement of serum total calcium [9]. The stability of blood calcium levels allows the non-recurrence of seizures and, thus, the weaning of anti-epileptic drugs.

Our patient was started with valproate, and the outcome was good after calcium-vitamin D therapy. We progressively reduced the doses of anti-epileptic drugs. We have not stopped the treatment yet, and the patient is currently on very low doses of valproate. We attempted to withdraw anti-epileptic drugs gradually in two years under a normal EEG.

## Conclusions

Hypoparathyroidism is an uncommon disease, with symptoms mainly associated with hypocalcemia in children. This case illustrates the importance of performing, as a first intention, an electrolyte profile, especially serum phosphocalcium, in patients with new-onset seizures. This is mandatory to detect any metabolic abnormality and allow an early diagnosis and appropriate management of this pathology.
